# Glial Cells of the Central Nervous System: A Potential Target in Chronic Prostatitis/Chronic Pelvic Pain Syndrome

**DOI:** 10.1155/2023/2061632

**Published:** 2023-11-13

**Authors:** Yongfeng Lao, Zewen Li, Yanan Bai, Weijia Li, Jian Wang, Yanan Wang, Qingchao Li, Zhilong Dong

**Affiliations:** ^1^Second Clinical Medical College, Lanzhou University, Lanzhou, China; ^2^Department of Urology, Lanzhou University Second Hospital, Lanzhou, China; ^3^Laboratory Medicine Center, Lanzhou University Second Hospital, Lanzhou, China

## Abstract

Chronic prostatitis/chronic pelvic pain syndrome (CP/CPPS) is one of the most common diseases of the male urological system while the etiology and treatment of CP/CPPS remain a thorny issue. Cumulative research suggested a potentially important role of glial cells in CP/CPPS. This narrative review retrospected literature and grasped the research process about glial cells and CP/CPPS. Three types of glial cells showed a crucial connection with general pain and psychosocial symptoms. Microglia might also be involved in lower urinary tract symptoms. Only microglia and astrocytes have been studied in the animal model of CP/CPPS. Activated microglia and reactive astrocytes were found to be involved in both pain and psychosocial symptoms of CP/CPPS. The possible mechanism might be to mediate the production of some inflammatory mediators and their interaction with neurons. Glial cells provide a new insight to understand the cause of complex symptoms of CP/CPPS and might become a novel target to develop new treatment options. However, the activation and action mechanism of glial cells in CP/CPPS needs to be further explored.

## 1. Introduction

Prostatitis is one of the most common urological diseases in adult men. According to the National Institutes of Health (NIH) classification, there are four types of prostatitis, namely, type I (acute bacterial prostatitis (ABP)), type II (chronic bacterial prostatitis (CBP)), type III (chronic prostatitis/chronic pelvic pain syndrome (CP/CPPS)), and type IV (asymptomatic inflammatory prostatitis (AIP)) [1]. CP/CPPS accounts for more than 90% of prostatitis cases while the incidence is as high as 10%–14% [2, 3]. The possible risk factors of CP/CPPS included urethritis, uncontrolled sexual intercourse, frequent masturbation, fixed body position (especially prolonged straddling and sitting), alcohol abuse, lifestyle changes, prolonged urinary retention, and stress and anxiety [1]. Clinical symptoms of CP/CPPS are complex and varied, and current clinical guidelines classify them into four major clinical symptom clusters: pelvic pain symptoms, lower urinary tract symptoms, psychosocial symptoms, and sexual dysfunction symptoms [1, 4]. The symptoms of CP/CPPS usually last for 3–6 months or longer [5, 6]. Current common clinical treatments include alpha-adrenergic blockers, antibiotics, pain medication, specialist physiotherapy, phytotherapy, and antidepressants, but these options are often ineffective due to the complexity of CP/CPPS symptoms [7]. Therefore, understanding the mechanisms involved in causing the complex clinical symptoms of CP/CPPS might help us find more effective therapeutic targets.

Glial cells, which play an important role in maintaining the stability and functional integrity of the central nervous system (CNS), constitute approximately half of all neural cells in the mammalian CNS [8]. There are mainly three types of glial cells in the CNS: microglia, astrocytes, and oligodendrocytes [9]. Over the past few decades, the function of different glial cells in physiological and pathological states has been explored deeply and widely. As the smallest glial cell in the CNS, microglia hold a vital role in the formation of neuronal circuits and neuronal survival [10], programmed cell death of neurons and removal of cellular debris to maintain normal neurological function [11], and modulation of synaptic function [12–14]. Microglia can also act as antigen-presenting cells because microglia express phagocytic and endocytic receptors that allow them to capture antigens [15]. Of all types of glial cells, astrocytes are the most widely distributed in the CNS. Astrocytes are closely involved in neuronal survival and synaptic function [16–18]. Also, the ion homeostasis (potassium homeostasis, chloride homeostasis, extracellular calcium homeostasis, and pH homeostasis) and neurotransmitter homeostasis in the CNS are associated with the normal function of astrocytes [17, 19–21]. Furthermore, astrocytes have a close relationship with the blood vessels in the brain because their terminal pedunculated processes completely wrap around all the cerebral vessels. Thus, astrocytes play an important role in mediating functional brain congestion [22]. Oligodendrocytes are discovered throughout the gray and white matter of the CNS. The most important function of oligodendrocytes is the formation of myelin sheaths [23, 24]. In addition, in the gray matter, a small proportion of oligodendrocytes are referred to as “perineuronal” satellite cells, which do not form myelin but are closely connected with the cell bodies of neurons, suggesting that there is an interdependent relationship. Some oligodendrocytes are closely associated with small blood vessels and are therefore called “perivascular satellites” [25]. Until now, new functions of CNS glial cells in different diseases and conditions are still being discovered.

In recent years, glial cells were found to be involved in the occurrence and progression of various symptoms such as pain, psychosocial symptoms, and lower urinary tract symptoms through multiple pathways [26–32]. It indicated that glial cells might be the basis for the multisystemic complex clinical symptoms of CP/CPPS. The relationship between glial cells and CP/CPPS has already drawn a lot of attention. Abnormal activation of glial cells in the spine and brain has been found in experimental autoimmune prostatitis (EAP) rat models [33–35]. To better understand the role of glial cells in CP/CPPS, this narrative review extensively evaluated relevant literature to summarize current research progress and focus on the significance of three types of glial cells: microglia, astrocytes, and oligodendrocytes, in the pathogenesis of CP/CPPS.

## 2. Overview of Glial Cells

**Figure 1:**
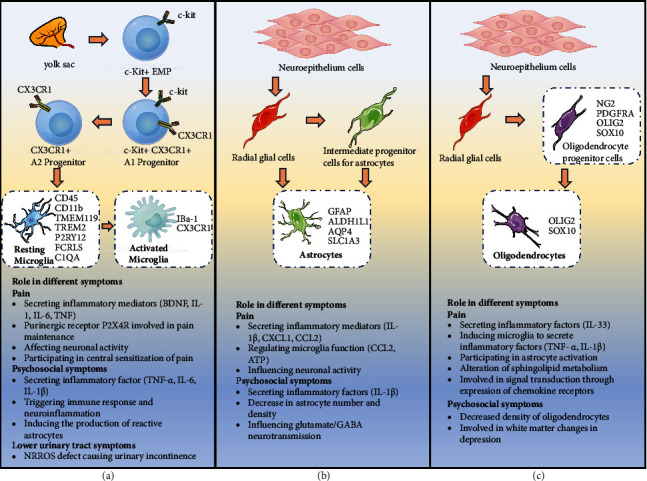
Differentiation process of glial cells and general function of glial cells in prostatitis-related symptoms (pain, psychosocial symptoms, and lower urinary tract symptoms). (a) Microglia originate in the yolk sac. At first, c-kit^+^ erythromyeloid progenitor (EMP) cells develop, mature, and expand in the yolk sac, and form c-kit^+^ CX3CR1^+^ A1 progenitor subsequently. CX3CR1^+^ A2 myeloid progenitor cells are derived from c-kit^+^ CX3CR1^+^ A1 progenitors and finally differentiate into microglia in the brain. Multiple markers have been found to specially recognize microglia (CD45, CD11b, TMEM119, TREM2, P2RY12, FCRLS, and C1QA) while IBa-1 and CX3CR1 are widely used markers of activated microglia in pathology studies. (b) Astrocytes originate from neuroepithelium cells. Neuroepithelial cells further transform into radial glial cells while radial glial cells can divide asymmetrically to produce different types of intermediate progenitor cells. Both radial glial cells and intermediate progenitor cells can differentiate into astrocytes. GFAP, ALDH1L1, AQP4, and SLC1A3 are common astrocyte markers. (c) Oligodendrocytes also originate from neuroepithelium cells. Radial glial cells derived from neuroepithelium cells can differentiate into oligodendrocyte progenitor cells (specific marker: NG2, PDGFRA, OLIG2, and SOX10). Oligodendrocytes are produced by oligodendrocyte progenitor cells. OLIG2 and SOX10 are the specific markers of mature oligodendrocytes.

In this part, we summarized the differentiation process and molecular markers of the three types of glial cells as shown in Figure 1.

### 2.1. Microglia

#### 2.1.1. Development and Differentiation

Microglia participate in the regulation of CNS function as resident immune cells in the CNS. The phenotypic similarity of microglia to peripheral blood monocytes/macrophages and dendritic cells makes it easier to accept the concept of their myeloid origin [36]. In recent years, an increasing number of studies have demonstrated that microglia originate in the yolk sac and grow in the CNS [36, 37]. Erythromyeloid progenitors (EMP) develop and differentiate into myeloid precursor cells in the yolk sac. Myeloid precursor cells mature, expand, and migrate, staying in different regions of the brain and eventually differentiating into microglia [38] (Figure 1).

#### 2.1.2. Molecular Markers

CD45 and CD11b are the most common cell surface markers of microglia [39]. It has also been reported that microglia specifically express the cell surface marker transmembrane protein 119 (TMEM119) [40]. In addition, many genes, including TREM2, P2RY12, FCRLS, and C1QA could also be used to identify microglia. These markers are located in the plasma membrane or outside the cell and are responsible for the rapid activation and secretion function of microglia [41]. In pathology studies, the ionized calcium-binding adapter molecule 1 (IBa-1) and chemokine receptor 1 (CX3CR1) are widely used markers of activated microglia [42, 43].

### 2.2. Astrocytes

#### 2.2.1. Development and Differentiation

Astrocytes are classical neuronal cells that originate from universal neural progenitors, which are radial glial (RG) cells derived from the neuroepithelium [44]. Early developing neuroepithelial cells divide symmetrically to produce more neuroepithelial cells and further transform into RG cells. Then RG cells divide asymmetrically to produce different types of intermediate progenitor cells, which occur in the subventricular zone [19]. Among them, intermediate progenitor cells for astrocytes further develop into astrocytes. At the end of embryonic development, most RG cells' ventricular processes degenerate and begin to detach from the apical part and transform into astrocytes [44]. However, the astrocytes produced during the embryonic period account for only a small fraction of the astrocytes in the central nervous system. The generation of astrocytes after birth is mainly associated with the symmetrical division of differentiated astrocytes in the central nervous system [45]. It has also been shown that the direct conversion of RG cells to astrocytes persists after birth [46]. In addition, there is evidence that astrocytes could be derived from oligodendrocyte progenitor cells (OPCs) in the ventral forebrain under some pathological conditions [47–49].

#### 2.2.2. Molecular Markers

Glial fibrillary acidic protein (GFAP) and aldehyde dehydrogenase 1 family member L1 (ALDH1L1) are the two most commonly used markers in astrocyte research [50, 51]. In addition, the aquaporin AQP4 and SLC1A3, which encode a high-affinity glutamate transporter protein, are also common astrocyte markers [41].

### 2.3. Oligodendrocytes

#### 2.3.1. Development and Differentiation

Oligodendrocytes are myelin-forming cells of the CNS. Oligodendrocytes are produced by oligodendrocyte progenitor cells (OPCs) under the control of multiple factors, including neurotransmitters and other neurogenic factors [23]. OPCs are also referred to as “NG2 cells” because they express NG2 proteoglycans on their surface [47, 52]. OPCs arise from the ventricular germinal zone of the embryonic neural tube and are generated by the asymmetric division of RG cells [52]. One of the daughter cells generated by RG cells' asymmetric division loses its connection to the ventricular and pial surfaces. Subsequently, they proliferate rapidly and migrate in various directions. These ventral-derived OPCs migrate into the developing gray and white matter before differentiating into myelin-forming oligodendrocytes [52]. Unlike most progenitor cells, OPCs remain abundant in the adult central nervous system and are present throughout human life [47, 52]. OPCs in adults retain the ability to generate new oligodendrocytes, allowing rapid regeneration of myelin sheaths that may be lost in normal aging or disease [53].

#### 2.3.2. Molecular Marker

The expression of molecular markers of oligodendrocytes varies at different stages of development. OPCs expressed NG2, platelet-derived growth factor receptor *α* (PDGFRA), transcription factors OLIG2 and SOX10, and other molecular markers. When OPCs began to differentiate into oligodendrocytes, NG2 and PDGFRA were rapidly downregulated, but OLIG2 and SOX10 continued to be expressed in mature oligodendrocytes [52].

## 3. Association of Glial Cells with Prostatitis-Related Symptoms

In this part, we summarized the roles of glial cells in prostatitis-related symptoms. Three symptoms were involved: pain, psychosocial symptoms, and lower urinary tract symptoms. The association between sexual dysfunction and glial cells was seldom studied.

### 3.1. Glial Cells and Pain

#### 3.1.1. Microglia

Microglia play an important role in different stages of pain, including pain occurrence and maintenance, as well as nociceptive signaling and nociceptive sensitization (Figure 1).

*(1) Occurrence of Pain*. Under normal physiological conditions, microglia are considered “dormant” cells of the CNS, but they are not static. Microglia constantly monitor the surrounding environment through their ramified processes and are involved in pain regulation when the CNS is stimulated by trauma, ischemia, and infection, which is a key factor in the onset of pathological pain [54]. Activated microglia will produce some nociceptive modulators or pain-increasing substances, such as BNDF, IL-1, IL-6, and TNF, which cause pain [27].

*(2) Maintenance of Pain*. Microglia mediate the transition from acute to chronic pain through the expression of the purinergic ATP receptor P2X4R and the release of BDNF from P2X4R^+^ microglia [35, 55]. Toyomitsu et al. demonstrated that C-C motif ligand 2 (CCL2) could promote the expression of P2X4R on cell surface expression of microglia, regulating the maintenance of chronic pain through the P2X4R receptor pathway [56].

*(3) Nociceptive Signaling and Nociceptive Sensitization*. The purinergic ATP receptor P2X7 plays an important role in the secretion of pro-inflammatory cytokines mediated by microglia activation [57]. P2X7 receptors are quiescent under normal physiological conditions but are activated under pathological conditions. Activated P2X7 receptors are involved in pain transmission, significantly increase secretion of pro-inflammatory cytokines, and are associated with calcium-related signaling [58, 59]. Previous studies have revealed that in rats with inflammatory pain, visceral pain, and neuropathic pain, local or intraperitoneal injection of P2X7 antagonists inhibited nociceptive hyperalgesia. Meanwhile, neuropathic hypersensitivity to mechanical or thermal stimuli was lost in P2X7 receptor-deficient mice [60].

#### 3.1.2. Astrocytes

*(1) Occurrence and Maintenance of Pain*. Astrocytes are the most numerous glial cells, accounting for 20–60% of the brain cell population, and their numbers vary markedly by species and brain region [61]. Recent studies on astrocytes have shown that their cell membranes contain many ion channels and receptors for neurotransmitters, and they are capable of producing and releasing neuroactive substances, which may play an important role in the development and maintenance of some chronic pain [62]. Connexin 43 (Cx43) is a major connexin expressed in astrocytes that enhances the expression and secretion of cytokines and chemokines in certain types of cells and also acts as a non-ligated hemichannel that releases small molecules and ions. It was found that activated astrocytes could release various inflammatory mediators and neuromodulators, such as cytokines (IL-1*β*) and chemokines (CXCL1 and CCL2), through upregulation of Cx43, to enhance and prolong persistent pain states [63–65] (Figure 1).

#### 3.1.3. Oligodendrocytes

*(1) Occurrence of Pain*. IL-33 is predominantly expressed by oligodendrocytes in the central nervous system [66]. IL-33 from oligodendrocytes may activate ST2 receptors on spinal microglia and activate intracellular signaling pathways such as phosphatidylinositol 3 kinase (PI3K), mammalian target of rapamycin protein (mTOR), mitogen-activated protein kinase (MAPK), and nuclear factor kappa-B (NF-*κ*B), which then release pro-inflammatory cytokines such as TNF-*α* and IL-1*β*, leading to neuropathic pain [67].

*(2) Nociceptive Signaling and Nociceptive Sensitization*. Sphingolipid metabolism is altered in oligodendrocytes following stimuli such as inflammation, which is associated with astrocyte activation, pro-inflammatory cytokine release, neuronal sensitization, and increased nociception, although the specific targets of sphingolipid metabolites on astrocytes and neurons are not known [68]. MOG, a high-affinity site for a nerve growth factor (NGF) and located on the outer surface of myelin oligodendrocytes and the outer layer of myelin sheath in the central nervous system, may play an important role in oligodendrocyte-associated pain [69, 70]. The expression of MOG is a protective mechanism that depletes excess NGF, thereby preventing the abnormal neuropathic pain triggered by peripheral nerve injury [70]. Also, in some diseases, disruption of MOG may cause dysfunction of the NGF, leading to an increase in pain [68]. Oligodendrocytes express chemokine receptors, such as CXCR1 and CXCR2 [71], while chemokines such as CXCL1 have been shown to play an important role in nociceptive signaling [63, 64]. However, the relationship between oligodendrocytes and chemokines in pain needs to be further elucidated (Figure 1).

#### 3.1.4. Glial Cell-Neuron Interactions and Pain

In the CNS, there are extensive connections and interactions between glial cells and neurons, playing a key role in the onset and development of pain. Activation of either microglia or astrocytes promotes activation of the other, thus creating positive feedback [72, 73]. CCL2 from astrocytes can promote microglia proliferation in vitro, and in the absence of CCL2 in astrocytes, the aggregation of inflammatory cells in the CNS decreases, as well as the number of microglia decreases [74]. It was reported that CX43 in astrocytes could control the release of ATP which had a critical role in the activation of spinal microglia [64]. Actually, many neuroactive substances released by activated microglia and astrocyte in a sustained positive feedback manner are key regulators of pain sensitivity state [73]. Some researchers have demonstrated that microglial-derived BDNF could increase the excitability of spinal cord neurons through a variety of mechanisms, including diminished dorsal horn inhibition, enhanced dorsal horn excitation, and failure of descending inhibition, thereby participating in the generation and maintenance of centrally sensitized pain [75]. Additionally, activated spinal microglia produce inhibitory synaptic modulators reducing GABAergic synaptic strength and modulating glycinergic synaptic neurotransmission, thereby attenuating pain inhibition [29, 76]. Astrocytes are more dispersed and play a local integrative unit role, controlling the activity of other cells in their area and acting as a bridge between synaptic and non-synaptic communication. Astrocytes dynamically regulate synaptic information transfer [30].

### 3.2. Glial Cells and Psychosocial Symptoms

Psychosocial symptoms are associated with abnormalities of brain cell function, especially of glial cells [32] (Figure 1).

#### 3.2.1. Microglia

It was reported that the immunoreactivity of microglia was higher in the cortex of patients with depression, and positron emission tomography revealed enhanced microglia activation during major depressive episodes [77]. Microglia sense depression-related stressors and trigger the immune responses and neuroinflammation that lead to the development of depression [78]. Animal experiments provide more direct evidence for the role of microglia in depression. Activation of microglia was observed in the rodent models of LPS-induced depression [78], and the activation of indoleamine 2,3-dioxygenase (IDO) in microglia was found critical for the development of depression-like symptoms and LPS-induced microglia activation [79, 80]. Treatment with the microglia inhibitor minocycline attenuates LPS-induced depression-like symptoms [81]. Activation of microglia in depression occurs primarily in brain regions that process emotional stimuli and set emotional responses [32]. Some researchers have found activation of microglia and increased expression of the pro-inflammatory cytokine IL-1*β* in the amygdala, an important brain region regulating emotional and pain responses, in a rat model of depression [82]. Also, microglia-derived pro-inflammatory factors, such as TNF-*α*, IL-6, and IL-1*β*, are proven to be involved in the regulation of depression by modulating neuronal function [78]. It has also been shown that microglia activation and induction of inflammation in the hippocampus of rats may be associated with the development of depression [31].

#### 3.2.2. Astrocytes

Astrocyte pathology is a prominent feature of mood disorders [83]. Several components critical to astrocyte function were found to downregulate in studies exploring the abnormal gene expression in the brain tissue of patients with depression including SLC1A3, GLUL, GFAP, and S100 calcium-binding protein [84, 85]. The reduction of astrocyte number and density was reported in chronic stress and maternal deprivation model of depression in rodents [83]. Also, the number and density of astrocytes were observed to be reduced in the cortical regions of depression patients while selective pharmacological inhibition of glial cells in the rat prefrontal cortex could produce depression-like behavior [86]. Depression caused by astrocyte defects may be associated with the altered neurotransmission of glutamate/GABA since astrocytes are involved in glutamate uptake, metabolism, and recycling [87].

#### 3.2.3. Oligodendrocyte

Oligodendrocyte abnormalities, including reduced oligodendrocyte density and reduced expression of key oligodendrocyte-associated and myelin-associated genes, were found in regions such as the prefrontal cortex and amygdala in depressed patients [88–90]. miR-21 was highly expressed in mature oligodendrocytes of white matter and gray matter in the mouse CNS while it was significantly reduced in the white matter near the orbitofrontal cortex in subjects with depression [91]. Further exploration showed that miR-21 was correlated with mRNA of myelin proteins, astrocytic GFAP, and oligodendrocyte-associated transcription factors while the specific mechanism of miR-21 in white matter alterations in depression still should be further investigated [91].

### 3.3. Glial Cells and Lower Urinary Tract Symptoms (LUTS)

The abnormal neurological function is the common non-obstructive cause of bladder dysfunction (urinary outflow obstruction), which can result from abnormal neuroglial cell function [92]. NRROS is a leucine-rich repeat transmembrane protein in the endoplasmic reticulum that has an important role in the maintenance of normal neural function. It is preferentially expressed in myeloid cells, mainly microglia and non-substantial perivascular macrophages in the CNS [93]. NRROS is essential for the development and function of microglia and astrocytes while deficiency of NRROS will lead to the lack of normal microglia and astrocyte functional defects and shortened lifespan [94]. It was reported that NRROS^−/−^ mice display astrogliosis and lack normal CD11b^hi^CD45^lo^ microglia while around 90% of mice develop urinary incontinence and 60% develop urogenital prolapse after 12 weeks of age [94] (Figure 1).

## 4. Glial Cells in CP/CPPS

**Figure 2:**
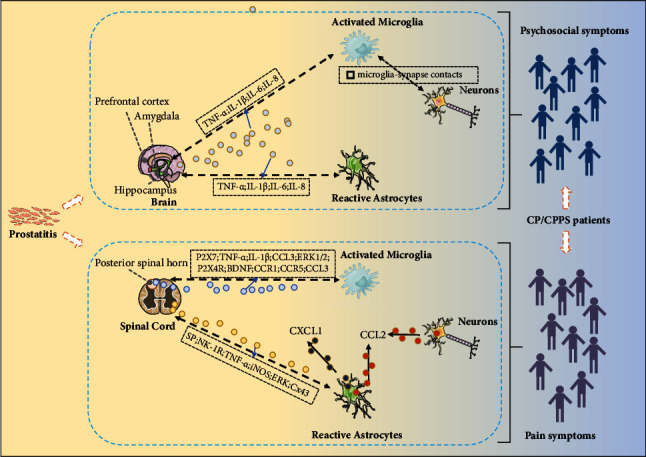
Schematic diagram of the potential function mechanism of glial cells in CP/CPPS. The black solid lines represent direct action or influence, the black dashed lines represent potential interactions, and the colorful circles represent substances that mediate potential interactions or are directly secreted by glial cells/neurons. Psychosocial symptoms in CP/CPPS may be associated with activated microglia, reactive astrocytes, and potential microglia-neurons interaction in the hippocampus of the brain. Microglia and astrocytes may function through or be activated by TNF-*α*, IL-1*β*, IL-6, and IL-8. Activated microglia may interact with neurons by microglia-synapse contacts to regulate psychosocial symptoms in CP/CPPS. Pain symptoms in CP/CPPS may be associated with activated microglia, reactive astrocytes, and neurons in the spinal cord. Microglia may function through or be activated by P2X7, TNF-*α*, IL-1*β*, CCL3, ERK1/2, P2X4R, BDNF, CCR1, CCR5, and CCL3. Astrocytes may function through or be activated by SP, NK-1R, TNF-*α*, iNOS, ERK, and Cx43. CXCL1 expressed in reactive astrocytes may be a potential target for pain syndromes in CP/CPPS. CCL2 expressed in reactive astrocytes and neurons may regulate pain symptoms in CP/CPPS.

Currently, evidence reporting the association between glial cells and CP/CPPS focuses on microglia and astrocytes. We summarized the potential role and mechanism of these two glial cells in CP/CPPS based on existing evidence in Figure 2.

### 4.1. Microglia

#### 4.1.1. Pain

The main nerve innervating the prostate gland is the pelvic nerve, which originates from the L5-S2 spinal cord [95]. Neurogenic inflammation was found to exist in the L5-S2 spinal cord of experimental autoimmune prostatitis (EAP) rats, along with an increased number of microglia and in a highly activated state (high expression of IBa-1) [96]. Further exploration revealed elevated P2X7 expression in the posterior horn of the L5-S2 spinal cord and increased TNF-*α* and IL-1*β* secretion, which is associated with the activation of microglia [96]. Similarly, Wong et al. also found microglia activation in the sacral spinal cord (S1–S4) in EAP-induced mice which was closely associated with increased levels of CCL3, IL-1*β*, IBa-1, and ERK1/2 phosphorylation [35]. Also, microglial activation in mice with prostatitis resulted in increased expression of two molecular markers associated with chronic pain in the spinal cord: P2X4R and BDNF [35, 55]. EAP-induced changes in spinal microglia might be restricted to the sacral segment of the spinal cord since differentially expressed CCL3 and its receptors CCR1 and CCR5 could be observed in the sacral segment, not the thoracic segment [35]. CCL3 in the spinal cord was proven to be involved in the development of pain by inducing the expression of the pro-inflammatory cytokine IL-1*β* [97]. Anyway, microglia might play a vital role in prostatitis-induced pain by secreting or regulating some pain-related substances.

#### 4.1.2. Psychosocial Symptoms

As psychosocial symptoms often appeared in patients with CP/CPPS, depression- and anxiety-like behaviors are also observed in EAP-induced mice as well as learning-memory impairment [33, 98]. Du et al. reported that activated microglia (increased levels of IBa-1) were increasing in the hippocampus of EAP mice with depression- and anxiety-like behavior [98]. Then, increasing expressions of pro-inflammatory mediators including TNF-*α*, IL-1*β*, IL-6, and IL-8 were found in the hippocampus which might be due to the actication of microglia [98]. Some neurobiological alterations might lead to behavioral changes in EAP mice such as abnormal glutamate metabolism and decreased serotonin [98]. However, the association between microglia activation of such neurobiological alterations needs to be further investigated. Additionally, Du and colleagues further discovered a reduction of dendritic complexity and spine density in the hippocampus as well as the structural changes in hippocampal synaptic plasticity which could describe the learning and memory decline of EAP mice [33]. And, more microglia were found perineuronal located and appeared to enwrap the NeuN-labeled neuronal perikarya tightly in the EAP mice, suggesting a regulatory role of microglia by more close microglia-synapse contacts [33].

### 4.2. Astrocytes

#### 4.2.1. Pain

Astrocytes have been proven to be involved in EAP-induced pain and potential targets for certain interventions in the past decade or so [99–102]. Zhang et al. found increasing activated astrocytes in the L6-S1 spinal dorsal horn of EAP rats where substance P (SP) was also elevated indicating a possible association [99]. Similarly, activated astrocytes were also found to increase in the L5-S2 spinal dorsal horn of EAP rats. Also, SP, NK-1R, TNF-*α*, and iNOS were elevated in the L5-S2 spinal dorsal horn [100]. It was reported that electroacupuncture could release pain symptoms of chronic prostatitis rats while inhibiting astrocyte activation and CXCL1 expressions in astrocytes in the L5-S2 spinal dorsal horn, indicating that CXCL1 in astrocytes might be a potential target for chronic prostatitis [101]. Additionally, Liu et al. detected elevated CCL2 expression of the lower dorsal horn of the spinal cord which was mainly from GFAP-positive astrocytes and NeuN-positive afferent neurons [103]. Elevated CCL2 might be involved in early macrophage recruitment in the dorsal root ganglion [103]. Furthermore, although microglia and astrocytes seemed to be both involved in EAP-induced pain, Deng et al. found that activated spinal astrocytes might be more crucial in maintaining EAP-induced persistent pain [34]. The authors observed increasing activated microglia (Iba-1 positive) and astrocytes (GFAP positive) in L5–S1 spinal cord dorsal horn at 2 weeks after model establishment while only increasing activated astrocytes were observed at 5 weeks [34]. Further mechanism exploration indicated that ERK, Cx43, and CXCL1 in the L6∼S1 lumber cord sections might be crucial for astrocyte activation and function [34].

#### 4.2.2. Psychosocial Symptoms

In a study focusing on EAP-induced depression-like behavior, increasing reactive astrocytes were observed in the hippocampus [98]. Although the authors attributed some abnormal pro-inflammatory mediators including TNF-*α*, IL-1*β*, IL-6, and IL-8 in the hippocampus to the activation of microglia, they could not rule out a link between reactive astrocytes and the pro-inflammatory mediators.

## 5. Discussion

As a common yet complex disease of the urinary system, CP/CPPS has been puzzling clinicians for a long time because pain may arise from the urogynecological, gastrointestinal, pelvic musculoskeletal, or nervous systems [104] and is often accompanied by other symptoms. Understanding pathophysiology of the complex multifactorial process of CP/CPPS may be vital to establishing effective treatments. As an important participant in several symptoms that overlapped with CP/CPPS-related symptoms and one of the earliest known pathological changes outside the genitourinary system, glial cells provide a unique perspective to approach the etiology of complex manifestations of CP/CPPS. In this narrative review, we reviewed the biological characteristics of glial cells and their general role in pain, psychosocial symptoms, and lower urinary tract symptoms which were commonly seen in CP/CPPS patients. Also, we summarized the research process of glial cells in CP/CPPS.

After review, two types of glial cells: microglia and astrocytes, were reported to be involved in pain and neuropsychiatric issues in CP/CPPS. For psychosocial symptoms in CP/CPPS, microglia and astrocytes in the brain may function through or be activated by TNF-*α*, IL-1*β*, IL-6, and IL-8 while activated microglia may interact with neurons by microglia-synapse contacts to regulate the psychosocial symptoms. For pain symptoms in CP/CPPS, microglia in the spinal cord may function through or be activated by P2X7, TNF-*α*, IL-1*β*, CCL3, ERK1/2, P2X4R, BDNF, CCR1, CCR5, and CCL3. Also, astrocytes in the spinal cord may function through or be activated by SP, NK-1R, TNF-*α*, iNOS, ERK, and Cx43. However, these preliminary shreds of evidence could not support the logical correlation between abnormal glial cells and the pathology of CP/CPPS, that is, how the glial cells are activated, or what abnormally activated glial cells bring to EAP-induced pathology and symptoms, etc. CXCL1 and CCL2 which have a wide range of functions in chronic pain [105, 106] were found abnormally expressed in reactive astrocytes or neurons in EAP-induced pain symptoms. They were the only two molecules that were reported to localize to specific cells indicating that these two molecules might be potential targets to regulate the astrocytes and neurons in CP/CPPS. Interestingly, Deng et al. reported that activated spinal astrocytes might be more crucial in the maintenance of EAP-induced persistent pain [34]. Actually, the initiation and maintenance of pain is an extremely complex process [107]. It was reported that the crosstalk process between microglia, astrocytes, and neurons held a key role in the maintenance of neuropathic pain while the microglia P2X4 receptor was the key target that could activate astrocytes and neurons later [108]. Deng's observation indicated potentially different roles and crosstalk of glial cells in CP/CPPS which required further confirmation. Furthermore, we should realize that difference from Deng's study might come from differences in the model because carrageenan was utilized to induce prostatitis pain [109] which was different from many other studies [98, 103]. Thus, the results should also be validated in the EAP model induced utilizing other common methods. Anyway, although current results of glial cells in CP/CPPS suggest an exciting potential to lift the veil of CP/CPPS, studies were still preliminary and remain elusive and long.

Notably, when studies explored the association between glial cells and EAP-induced pain, only the spinal cord was looked at, and it was not often the same spinal cord segment. Reversely, when considering psychosocial symptoms, only brain microglia were studied. In fact, these different symptoms often appear simultaneously [110, 111], indicating possibly simultaneous activation of CNS glial cells both in the brain and the spinal cord. Brain microglia have also a vital role in neuropathic pain while microglia in the spinal cord were involved in psychosocial symptoms too [112–114]. It suggests a direction for future research, that is, the spatiotemporal and functional connections of CNS glial cells in EAP-induced pathology. Furthermore, as shown in our review, various molecular markers were identified to recognize and distinguish different glial cells. However, the use precision of these molecular markers in CP/CPPS might be improved. IBa-1 was used for evaluating the activation of microglia in some studies [96, 98] while it was used to mark both microglia and macrophage activation in the spinal cord [103]. Actually, IBa-1 is highly expressed in both microglia and monocytes/macrophages [115, 116]. Therefore, activated microglia observed in the EAP model might not avoid bias from the macrophage. Some studies applied multiple staining to distinguish and identify activated microglia, for instance, TMEM119 was utilized for recognizing microglia while IBA-1 was used to screen the activated part [117–119]. Several studies focusing on the glial cells in CP/CPPS have also adopted the above strategy such as CD11b^+^IBa1^+^ or TMEM119^+^IBa1^+^ to screen microglia more precisely [35, 103]. Therefore, to avoid confusion between the roles of microglia and macrophages in CP/CPPS, more specific molecular markers should be utilized in the future to distinguish microglia (whether activated or not) from monocytes/macrophages combined with makers to mark the activation status of microglia.

The multifunction of glial cells provides a new perspective on understanding the complex symptoms and mechanisms of CP/CPPS, but there are still some important issues to be resolved in the future. Firstly, there is a lack of clinical evidence of glial dysfunction in CP/CPPS patients. Non-invasive detection of glial cells in the human body is difficult. Recently, a non-invasive diffusion-weighted magnetic resonance imaging (MRI) method was reported to image changes in glial morphology [120]. It is possible that in the future such emerging technology could help to find evidence of glial dysregulation in CP/CPPS patients. Secondly, as noticed, the role of glial cells in sexual dysfunction has not yet been studied; the same is true for the role of glial cells in LUTS. With the development of new tools such as the UPOINTS system to better distinguish the symptoms of CP/CPPS [121], clarifying the function of glial cells in various prostatitis-related symptoms could help understand their potential as novel targets for CP/CPPS. Thirdly, functions of glial cells in CP/CPPS may vary from each other, and there may be complex crosstalk between glial cells and neurons while these functions and interactions have temporal and spatial effects. But so far, we still know very little about their functions and crosstalk. In particular, oligodendrocytes do not seem to have received any attention from existing research. All in all, there is still a great deal of work to be done to promote the clinical transformation of glial cells in CP/CPPS in the future.

In conclusion, this narrative review comprehensively looked back at the research progress of glial cells in prostatitis-related symptoms and CP/CPPS. But there are still some important issues to think about and solve, namely, what is the function of oligodendrocytes and crosstalk between different glial cells and neurons, how do glial cells activate and function, whether other symptoms in CP/CPPS such as LUTS and sexual dysfunction are regulated by glial cells, etc. Anyway, glial cells show new ideas for the etiology and treatment of CP/CPPS, but there is still a long way off.

## Data Availability

The data used to support the findings of this study are available from the corresponding author upon request.
